# A Model for Growth of a Single Fungal Hypha Based on Well-Mixed Tanks in Series: Simulation of Nutrient and Vesicle Transport in Aerial Reproductive Hyphae

**DOI:** 10.1371/journal.pone.0120307

**Published:** 2015-03-18

**Authors:** Wellington Balmant, Maura Harumi Sugai-Guérios, Juliana Hey Coradin, Nadia Krieger, Agenor Furigo Junior, David Alexander Mitchell

**Affiliations:** 1 Departamento de Bioquímica e Biologia Molecular, Universidade Federal do Paraná, Cx.P. 19046 Centro Politécnico, Curitiba 81531–980, Paraná, Brazil; 2 Departamento de Engenharia Química e Engenharia de Alimentos, Universidade Federal de Santa Catarina, Cx.P. 476 Centro Tecnológico, Florianópolis 88040–900, Santa Catarina, Brazil; 3 Departamento de Engenharia Química, Universidade Federal do Paraná, Cx.P. 19011 Centro Politécnico, Curitiba 81531–980, Paraná, Brazil; 4 Departamento de Química, Universidade Federal do Paraná, Cx.P. 19081 Centro Politécnico, Curitiba 81531–980, Paraná, Brazil; University of Nebraska, UNITED STATES

## Abstract

Current models that describe the extension of fungal hyphae and development of a mycelium either do not describe the role of vesicles in hyphal extension or do not correctly describe the experimentally observed profile for distribution of vesicles along the hypha. The present work uses the n-tanks-in-series approach to develop a model for hyphal extension that describes the intracellular transport of nutrient to a sub-apical zone where vesicles are formed and then transported to the tip, where tip extension occurs. The model was calibrated using experimental data from the literature for the extension of reproductive aerial hyphae of three different fungi, and was able to describe different profiles involving acceleration and deceleration of the extension rate. A sensitivity analysis showed that the supply of nutrient to the sub-apical vesicle-producing zone is a key factor influencing the rate of extension of the hypha. Although this model was used to describe the extension of a single reproductive aerial hypha, the use of the n-tanks-in-series approach to representing the hypha means that the model has the flexibility to be extended to describe the growth of other types of hyphae and the branching of hyphae to form a complete mycelium.

## Introduction

Mathematical models for the growth of filamentous fungi can be classified into three groups, according to the scale at which the phenomena are described: tip-scale models, intermediate-scale models and macro-scale models [1]. Tip-scale models limit themselves to describing the phenomena occurring from a point about 100 μm behind the hyphal tip up to the apex of the tip itself; they typically focus on describing the shape and the extension rate of the tip, but not the production of vesicles in the sub-apical region. The classical tip-scale model is the vesicle supply center model, originally developed by Bartnicki-Garcia et al. [2], but recently updated by Tindemans et al. [3] to describe a more realistic mechanism for delivery of vesicles from the vesicle supply centre to the membrane at the tip. At the other extreme, macro-scale models describe the interaction between the fungus and the environment; they do not recognize individual hyphae, but rather represent the fungus in terms of overall densities [1]. Intermediate-scale models, which have also been referred to as “single colony scale” models [1], are situated between these two extremes of scale. These models describe the extension of hyphae and their branching to form a complex hyphal network, also known as a mycelium. They often describe these phenomena as depending on the absorption and intracellular transport of nutrients. These models may be formulated as “continuum models”, in which individual hyphae are not recognized as physical entities, but rather biomass and other variables are expressed as average concentrations in space [1]. Alternatively, they may be formulated as “discrete models”, in which fungal hyphae are described as occupying specific locations within the available space [1]. Such models can be used to generate simulated images of mycelial networks [1].

The current work focuses on discrete intermediate-scale models. Such models need to describe the phenomena involved in the extension of hyphal tips at an appropriate level, which should be neither too simple nor too detailed [4]. Although the mechanisms are still not fully understood, tip extension in fungi involves the following steps: (1) membrane-bound vesicles are produced from internal nutrients in a sub-apical region of the hypha and contain enzymes necessary for the extension of the cell wall; (2) these vesicles are transported along the cytoskeleton from the sub-apical region to the tip of the hypha by motor proteins; (3) a multicomponent complex rich in vesicles, the Spitzenkörper, is located at the apex of the tip; its suggested function is to direct the vesicles to the membrane; (4) the vesicles fuse at the tip [5,6,7]. These mechanisms result in a slow increase in the concentration of vesicles along the vesicle-producing region of the hypha (in the direction of the tip), with a marked increase in the final 10 μm or so [8].

Microscopic analyses indicate the existence of at least two groups of vesicles: macrovesicles (70–90 nm in diameter) and microvesicles (30–40 nm in diameter). It has been suggested that the macrovesicles carry the components of the amorphous part of the cell wall and extracellular enzymes for later secretion, while the microvesicles, also called chitosomes, contain chitin synthases, which will become integral proteins of the cell membrane [9,10]. When both groups of vesicles fuse to the tip membrane, their vesicular membranes become new hyphal membrane and the new cell wall is synthesized by the enzymes carried by the vesicles. Other types of vesicles probably exist [9,10].

The vesicles regulate important aspects of mycelium development [7]. The rate of hyphal extension depends on the rate of fusion of vesicles at the tip membrane. The direction of growth depends on the exact position of the membrane where fusion occurs, which itself is defined by the position of the Spitzenkörper. In addition, the formation of sub-apical branches is triggered by the accumulation of vesicles in a sub-apical region.

Despite the importance of vesicles, only a few discrete intermediate-scale models for fungal growth have described them. In fact, most intermediate-scale models describe the extension of the hypha as depending on the intracellular concentration of nutrients at the tip. The only discrete intermediate-scale models that describe the role of vesicles in hyphal extension are those of Prosser and Trinci [7], Yang et al. [11] and López-Isunza et al. [12].

The model of Prosser and Trinci [7], for the extension of a mycelium over a surface, describes spore germination, the production and convective transport of vesicles, the fusion of vesicles at the tip and the formation of septa. Also, branching occurs due to accumulation of vesicles behind the tip. The hyphal length is divided into small segments, with vesicles being produced in each segment, except for the last segment. In this last segment, vesicles are consumed for tip extension, following a Michaelis-Menten-type equation. The main drawback of this model is that it does not consider the intracellular concentration of nutrients and its influence on the rate of vesicle production.

The model of Yang et al. [11], developed for liquid fermentation, also describes the production and transport of vesicles and septation. As was the case with the model of Prosser and Trinci [7], vesicle production is not described as depending on the concentration of intracellular nutrients. Additionally, it is assumed that vesicles move by diffusion, whereas, in reality, they are actively transported along the cytoskeleton.

The most recent discrete intermediate-scale model to describe vesicles is that of López-Isunza et al. [12], which describes the extension of a single germ tube over a substrate surface, prior to the first branching. This model incorporates Michaelis-Menten-type expressions for the production and the consumption of vesicles, convective and diffusive transport of nutrients and vesicles along the length and radius of the germ tube, and diffusion of the nutrient in the extracellular medium. It is the only model to consider two populations of vesicles, with the macrovesicles being produced along the length of the germ tube and the microvesicles being produced only at the tip. The hyphal extension rate depends on the concentrations of both types of vesicle. This model has two key disadvantages. Firstly, vesicles are transported with the same convective velocity as the intracellular nutrient, whereas they should have different velocities as the transport mechanisms are different. Secondly, the use of partial differential equations to describe both axial and radial transport of nutrients and vesicles might be reasonable for describing extension of a single germ tube, but it would be untenable for modeling a hyphal network.

The aim of the current work was to develop a discrete intermediate-scale model of hyphal extension that not only describes the role of vesicles, but that can also be used as the basis for modeling the development of a hyphal network. We did this by representing the hypha as a series of well-mixed tanks (in other words, we used the “n-tanks-in-series approach”), and by using appropriate balance equations to describe the transport of nutrients and the production, transport and consumption of vesicles. The model was used to simulate experimental results from the literature for the growth of reproductive aerial hyphae of *Rhizopus oligosporus* [13], *Aspergillus giganteus* [14] and *Phycomyces blakesleeanus* [15]. A sensitivity analysis was then undertaken to identify the parameters that have the greatest influence on the tip extension rate. Suggestions are made as to how this model can be extended to describe different types of hyphae and the development of a mycelium in three dimensional space.

## Model

### Selection of the type of hypha to be modeled

**Fig 1:**
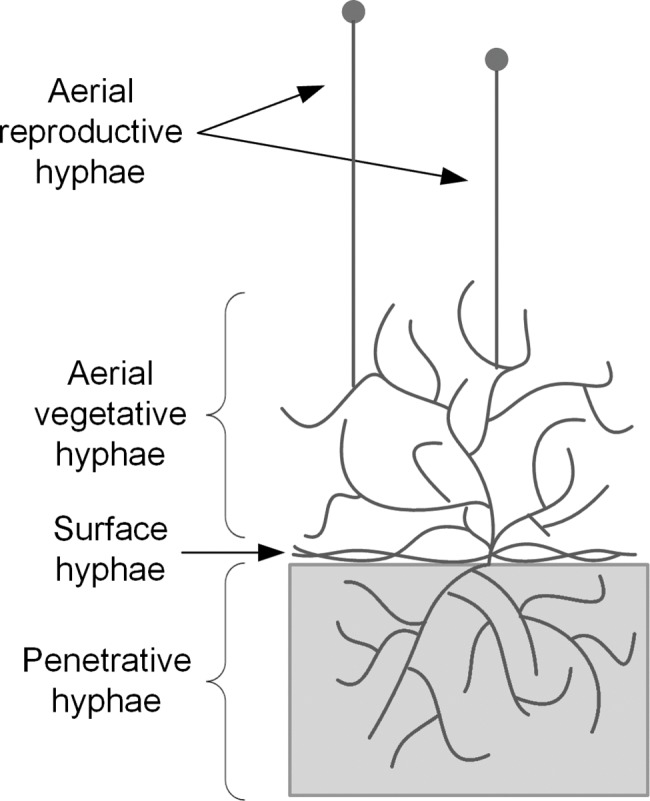
Simplified diagram of the growth of filamentous fungi on solid substrates. The different types of hyphae present are indicated.

Although all types of hyphae have important roles in the development of a fungal mycelium, we chose to model the extension of a reproductive aerial hypha. The spatial and physiological simplicity of the system (Fig. 1) allows the focus to be put on the role of vesicles in hyphal growth. If surface or penetrative hyphae were chosen, it would be necessary to include redirection of the hyphal tip, nutrient absorption through the membrane throughout the hyphal length, absorption and transport of O_2_ inside the hypha, production of extracellular enzymes, extracellular hydrolysis of polymers and extracellular diffusion of nutrients, O_2_ and enzymes. As discussed later, our modeling approach can be adapted later to include such phenomena, in order to describe mycelial development in a spatially heterogeneous system.

### Description of an aerial hypha

**Fig 2:**
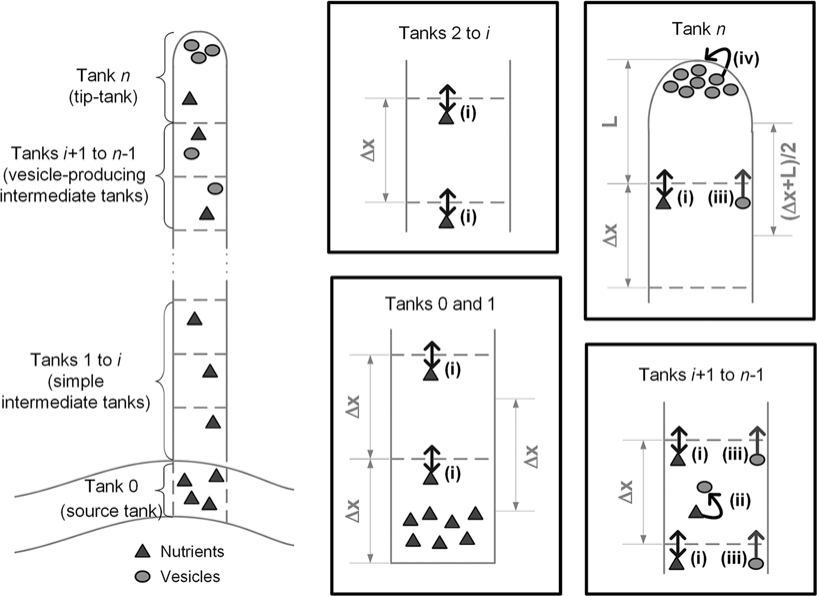
Description of a non-septate reproductive hypha as a series of well-mixed tanks. Phenomena are denoted by capital letters: (i) Nutrient is provided by the vegetative hypha (tank 0) at the base of the reproductive hypha (tanks 1 to *n*) and moves towards the tip (tank *n*) by diffusion between the tanks and convective flow of the cytoplasm. This convective flow is due to evaporation at the hyphal tip; (ii) In the vesicle-producing zone (tanks *i*+1 to *n*-1), nutrient is used to produce vesicles containing cell wall precursors; (iii) The vesicles move towards the tip with a fixed velocity (i.e. there is no diffusive contribution to their transport) that can be different from that of the cytoplasm; (iv) The tip (tank *n*) extends due to the absorption of vesicles.

The model describes the extension of the reproductive aerial hypha in a simplified manner (Fig. 2). The reproductive aerial hypha extends from a vegetative hypha. Its appearance is triggered by a complex cascade of metabolic signals [16] and it grows with few branches [17]. The phenomena involved in the extension of reproductive aerial hyphae have not been well studied, however, based on micrographs of *Aspergillus nidulans*, which show an accumulation of vesicles in the tip of a reproductive aerial hypha [18], we assume that the mechanisms involved are similar to those described for surface hyphae [19].

The carbon source for the growth of reproductive aerial hyphae is maltose (or trehalose in some fungi), which is obtained through hydrolysis of glycogen in the vegetative hyphae [17,19,20]. The carbon source and other nutrients are carried in the direction of the tip by an intracellular flow of cytoplasm (often called “cytoplasmic streaming”), which, in turn, is caused by the evaporation of water [21]. Since the lateral walls of aerial hypha are waterproofed by their hydrophobin coat, evaporation occurs exclusively at the tip [22,23].

The carbon source is used for the production of macrovesicles and microvesicles in the sub-apical region, corresponding to the vesicle-producing zone, with these vesicles being actively transported towards the tip [24]. The Spitzenkörper is present immediately behind the tip of the hypha. It receives the macrovesicles and microvesicles and directs them to be fused at the tip membrane [9].

### Simplifications

The model contains some simplifying assumptions: (a) The simulation starts when a short segment of the reproductive hypha has already been produced, thus the metabolic signals that trigger its appearance are not described in the model; (b) Vesicles are produced from a hypothetical nutrient, which subsumes not only the carbon source and but also other nutrients that supply elements such as nitrogen, phosphorus and sulfur; (c) The metabolic processes in the vegetative hypha are not described, rather the concentration of the hypothetical nutrient in the vegetative hypha is used as a boundary condition; (d) The vegetative hypha does not contribute to vesicle production; (e) Since the vesicle concentration remains relatively low along the hypha, except at the very tip [8], the energy required for vesicle transport towards the tip is assumed to be a relatively small part of overall cellular maintenance, such that the maintenance coefficient is independent of vesicle concentration; (f) Microvesicles and macrovesicles are not described separately, rather both are lumped together as “vesicles”; (g) The Spitzenkörper is not described explicitly, it is subsumed into the hyphal-extension reaction that occurs at the tip; (h) The intracellular flow of water associated with cytoplasmic streaming supplies the necessary amount of water to occupy the extending tip, while the excess is lost through evaporation through the tip.

### Representation of the system

The current model is designed to be fused with the model of Coradin et al. [25], a previous discrete intermediate-scale model that represents hyphae as stretches of adjacent cubes in space. Analogously, the current model treats hyphae as series of well-mixed cubic tanks (Fig. 2). The reproductive aerial hypha itself may contain up to three different types of tanks: simple intermediate tanks (i.e. that do not produce vesicles), vesicle-producing intermediate tanks, and a tip-tank. The tank of the reproductive aerial hypha that is furthest from the tip is attached to a source tank. This source tank represents the vegetative hypha and acts as a supply of the hypothetical nutrient, which, hereafter, will be referred to simply as “nutrient”.

Each functional compartment of the hypha is represented by a stretch of adjacent tanks, and the length of each compartment varies during the simulation, depending on the number of tanks that it contains. A key parameter is the maximum number of vesicle-producing tanks (*N*_*V*_), which determines the maximum possible length of the vesicle-producing zone of the hypha (λ). When the reproductive aerial hypha is shorter than or equal to *N*_*V*_ +1 tanks, all intermediate tanks produce vesicles. When the hypha is longer than *N*_*V*_ +1 tanks, the vesicle-producing tanks are the *N*_*V*_ tanks immediately behind the tip-tank.

Extension occurs by the addition of new tanks at the tip of the hypha. Potentially, this could be done by accumulating reserve material in the tip-tank (while maintaining a constant volume) and suddenly creating a whole new tip tank, which would become the new tip-tank, and changing the status of the previous tip-tank, which is now immediately behind the new tip-tank, to “vesicle-producing”. However, such an approach can easily lead to numerical instability [26]. In our model, the length of the tip-tank increases until it reaches double the length of a normal tank. When this happens, this “double-size” tip-tank is divided into two tanks of equal size (each equal to the length of a normal tank). After this division, the new “normal-sized” tank at the tip is designated as the tip-tank and the new “normal-sized” tank behind the tip is designated as a vesicle-producing intermediate tank.

### Model equations

Mass balances of nutrient (*ω*_*i*_) and vesicles (*ϕ*_i_) were written for each type of tank.

The first intermediate tank (tank number 1) is in contact with the source tank. The transport of nutrient to and from tank number 1 is through convection and diffusion. Inside the tank, nutrient is consumed for the formation of vesicles (with Michaelis-Menten-type kinetics) and for maintenance of the biomass. Thus, the mass balance for nutrient is given by:

$$\begin{array}{l} \frac{d\omega_{1}}{dt}=\frac{v}{\Delta x}(\omega_{0}-\omega_{1})+\frac{D}{\Delta x^{2}}(\omega_{0}-\omega_{1})\\ -\frac{D}{\Delta x^{2}}(\omega_{1}-\omega_{2})-\frac{1}{Y_{\phi }}\frac{k_{p}\omega_{1}}{K_{P}+\omega_{1}}-m\rho_{X} \end{array}$$ (1)

where *ω*_0_ is the concentration of nutrient in the source tank, *ω*_1_ is the concentration of nutrient in the first tank, *t* is the cultivation time, *v* is the intracellular convective velocity due to cytoplasmic streaming, Δ*x* is the length of a tank (and therefore the distance between centers of successive tanks), *D* is the diffusivity of nutrient, *Y*_*ϕ*_ is the yield coefficient for production of vesicles from nutrient, *k*_*p*_ is the maximum rate of vesicle production, *K*_*P*_ is the saturation constant for vesicle production, *m* is the maintenance coefficient, and *ρ*_*X*_ is the biomass dry weight per volume.

Since the first tank does not receive vesicles from the source tank, the balance on vesicles over the first tank is given by:

$$\frac{d\phi_{1}}{dt}=-\frac{\psi }{\Delta x}(\phi_{1})+\frac{k_{p}\omega_{1}}{K_{P}+\omega_{1}}$$ (2)

where *ϕ*_1_ is concentration of vesicles in the first tank and *ψ* is the velocity of active transport of vesicles.

The equations that describe the behavior of the intermediate tanks are similar to the equations for the first tank, because the same phenomena are present. The difference is that there is a flow of vesicles into these tanks. Therefore, the mass balances for nutrient and vesicles in tank *i* are, respectively:

$$\begin{array}{l} \frac{d\omega_{i}}{dt}=\frac{v}{\Delta x}(\omega_{i-1}-\omega_{i})+\frac{D}{\Delta x^{2}}(\omega_{i-1}-\omega_{i})\\ -\frac{D}{\Delta x^{2}}(\omega_{i}-\omega_{i+1})-\frac{1}{Y_{\phi }}\frac{k_{p}\omega_{i}}{K_{P}+\omega_{i}}-m\rho_{X} \end{array}$$ (3)

$$\frac{d\phi_{i}}{dt}=\frac{\psi }{\Delta x}(\phi_{i-1}-\phi_{i})+\frac{k_{p}\omega_{i}}{K_{P}+\omega_{i}}$$ (4)

Eqs. (1) to (4) apply to vesicle-producing tanks. When tank 1 or an intermediate tank loses the ability to produce vesicles, which happens when there are *N*_*V*_ tanks between it and the tip-tank, then the term describing the production of vesicles is set to zero in Eqs. (1) to (4).

In the tip-tank (tank *n*), the vesicles are consumed for the extension of the hypha. As described above, this tank has a variable length and, consequently, a variable volume. The velocity of extension of the tank (which corresponds to extension of the cell wall) is assumed to depend on the vesicle concentration according to a Michaelis-Menten-type relationship:

$$\frac{dL}{dt}=Y_{L}\frac{k_{c}\phi_{n}}{K_{C}+\phi_{n}}$$ (5)

where *L* is the length of the tip-tank, *Y*_*L*_ is the extension of hyphal length per mass of vesicle consumed, *k*_*c*_ is the maximum rate of vesicle consumption and *K*_*C*_ is the saturation constant for vesicle consumption.

The tip-tank only consumes vesicles, it does not produce them, and therefore maintenance is the only process that consumes nutrient in this tank. As a result, the balance on nutrient in the tip-tank is given by:

$$\frac{d\omega_{n}}{dt}=-\left(\frac{\omega_{n}}{L}\right)\frac{dL}{dt}+\frac{v}{L}\omega_{n-1}+\frac{D}{L\left(\frac{L+\Delta x}{2}\right)}(\omega_{n-1}-\omega_{n})-m\rho_{X}$$ (6)

where the first term on the right-hand side describes nutrient dilution due to the increase in the tank volume caused by tip extension. In the diffusion term of Eq. (6), the distance between the center of the tip-tank and the center of the preceding tank is not constant, but rather is equal to (*L*+Δ*x*)/2. The balance on vesicles in the tip-tank is given by:

$$\frac{d\phi_{n}}{dt}=-\left(\frac{\phi_{n}}{L}\right)\frac{dL}{dt}+\frac{\psi }{L}\phi_{n-1}-\left(\frac{1}{AL}\right)\frac{k_{c}\phi_{n}}{K_{C}+\phi_{n}}$$ (7)

where *A* is the cross-sectional area of the hypha. The first term on the right-hand side of Eq. (7) describes vesicle dilution due to the increase in the tank volume caused by tip extension.

When the double-sized tip-tank divides (i.e. when *L* = 2Δx), the two new normal-size tanks each have concentrations of nutrient and vesicles equal to the values for the double-sized tip-tank immediately before division.

### Boundary condition: The source tank

The source tank is numbered as tank 0. In this tank, there are no vesicles. For the simulations of growth of reproductive aerial hyphae of *Rhizopus oligosporus* and *Aspergillus giganteus*, which lasted up to 24 h, the concentration of nutrient in the source tank (*ω*_0_) was assumed to be constant. For the simulation of the growth of the sporangiophore of *Phycomyces blakesleeanus*, which lasted over 100 h, the concentration of nutrient fell during growth, according to the following mass balance:

$$\frac{d\omega_{0}}{dt}=-\frac{v\Delta x}{A_{0}}\omega_{0}-\frac{D}{A_{0}}(\omega_{0}-\omega_{1})$$ (8)

where *A*_*0*_ is the cross-sectional area of the source tank.

### Initial conditions, model parameters and numerical solution

Table 1 shows the values used for the parameters of the model. The length of the side of each cubic tank was chosen so as to give a cross-sectional area similar to that of the aerial hypha and was therefore different for each fungus: 10 μm for *Rhizopus oligosporus* [13], 40 μm for *Aspergillus giganteus* [27] and 150 μm for *Phycomyces blakesleeanus* [28]. Also, the maximum number of vesicle-producing tanks (*N*_*V*_) was different: 30 for *Rhizopus oligosporus*, 40 for *Aspergillus giganteus* and 5 for *Phycomyces blakesleeanus*. In this manner, the values of the maximum length of the vesicle producing zone (*λ*) were, respectively, 300 μm, 1600 μm and 750 μm. Such data are not available for aerial hyphae; these values are of the order of magnitude of values reported for the surface hyphae of a wide range of fungi [29]. In each case, the simulation was initiated with 3 tanks, the third tank being a normal-size tip-tank. Initially, the tanks contained neither nutrients nor vesicles (Table 1).

**Table 1:** Values of parameters and initial values of variables.

Symbol	Value (for parameters) or initial value (for variables)			Units	Description	Source
Parameters	*R*. *oligosporus*	*A*. *giganteus*	*P*. *blakesleeanus*			
*A*	1×10^-8^	1.6×10^-7^	2.25×10^-6^	dm²	Cross-sectional area of the hypha	[25, [27, [28]
*A*_*0*_			0.025	dm²	Cross-sectional area of the source tank	Calibrated
*D*	2.48×10^-4^	2.48×10^-4^	2.48×10^-4^	dm^2^ h^-1^	Diffusivity of nutrient inside the hypha	[30]
*k*_*c*_	2×10^-8^	3.2×10^-7^	4.5×10^-5^	g-vesicles h^-1^	Maximum rate of vesicle consumption	[29]
*K*_*C*_	400	1400	100	g-vesicles dm^-3^	Saturation constant for vesicle consumption	Calibrated
*k*_*p*_	1000	65	1000	g-vesicles dm^-3^ h^-1^	Maximum rate of vesicle production	Calibrated
*K*_*P*_	10	10	10	g-nutrient dm^-3^	Saturation constant for vesicle production	Calibrated
*m*	1.8×10^-3^	1.8×10^-2^	1.8×10^-2^	g-nutrient g-biomass^-1^ h^-1^	Maintenance coefficient of the hypha for nutrient	[31]
*ω*_0_	5	60	40	g-nutrient dm^-3^	Concentration of nutrient in the source tank	[17]
*v*	0.026	0.0236	0.15	dm h^-1^	Convective velocity inside the hypha	[32]
*Y*_*L*_	1×10^6^	6.25×10^4^	4.44×10^3^	dm g-vesicles^-1^	Extension of hyphal length per mass of vesicles consumed	[33]
*Y*_*φ*_	0.5	0.5	0.5	g-vesicles g-nutrient^-1^	Yield coefficient for production of vesicles from nutrient	[34]
Δ*x*	1×10^-4^	4×10^-4^	1.5×10^-3^	dm	Length of the side of each cubic tank	[[25], [27],[28]]
*N*_*v*_	30	40	5		Maximum number of tanks that the vesicle-producing zone can contain	[29]
*λ*	300	1600	750	μm	Maximum possible length of the vesicle-producing zone	[29]
*ρ*_*X*_	100	100	100	g-biomass dm^-3^	Biomass dry weight per volume	[33]
*ψ*	0.05	0.007	0.75	dm h^-1^	Velocity of active transport of vesicles inside the hypha	[35]
Variables						
*L*	1×10^-4^	4×10^-4^	1.5×10^-3^	dm	Length of the tip-tank	
*ω*_*i*_	0	0	0	g-nutrient dm^-3^	Concentration of nutrient in tank *i*	
*n*	3	3	150	tanks	Number of tanks present in the hypha	
*ϕ*_*i*_	0	0	0	g-vesicles dm^-3^	Concentration of vesicles in tank *i*	

The value for *Y*_*ϕ*_ is for growth of *Rhizopus oligosporus* [34]. *Y*_*L*_ was then calculated by assuming that all vesicles formed are consumed for tip extension and that the mass of biomass formed is equal to the mass of vesicles consumed. The values differ for the different fungi due to the differences in hyphal diameter (the larger the diameter the shorter the length of hypha formed per gram of vesicles consumed).

In each case, the model was calibrated by adjusting it to experimental results that have previously been published for reproductive aerial hyphae. In the case of *Rhizopus oligosporus*, Nopharatana et al. [13] obtained confocal microscopy images in which the maximum heights of hyphae above the substrate surface were 1000 μm at 16 h and 3300 μm at 40 h, with the reproductive hyphae being present only in the image corresponding to 40 h. In other words, the aerial hyphae extended 2300 μm in 24 h. In the case of *Aspergillus giganteus*, Trinci and Banbury [14] obtained detailed data for the length of “tall conidiophores” over a 21-h period. In both these cases, the period simulated corresponds to hyphal extension before the formation of conidia or sporangia. In the case of *Phycomyces blakesleeanus*, Gruen [15] obtained detailed data for the length of a sporangiophore over a 130-h period. However, we simulated only the final stage of growth, which corresponds to extension of the sporangiophore after the sporangium has already been formed. Gruen [15] reports this stage as starting at 20 h of cultivation, which corresponds to zero time in our simulation. Different sets of parameters were adjusted for each fungus, allowing for their morphological and physiological differences (Table 1).

After each simulation, it was checked whether the extension rate of the hypha was lower than the intracellular convective velocity (thereby confirming assumption (h) in section 2.3). This condition was satisfied for all simulations except for the artificial case of *v* = 0.

For the sensitivity analysis, which was carried out using the model for growth of the sporangiophore of *Rhizopus oligosporus*, the effect of each parameter was evaluated by calculating the percentage variation in the “hyphal length at 24 h” (denoted *F*) as follows:

$$F=\frac{y_{\max}-y_{\min}}{y_{\min}}\times 100\%$$ (9)

where *y*_max_ is the maximum “length at 24 h” obtained and *y*_min_ is the minimum “length at 24 h” obtained, when varying the parameter over the interval specified in Table 2.

The system of differential equations was solved using the FORTRAN subroutine DASSL [36], which uses a backward differentiation algorithm. The algorithm used for tank indexing and attribution of each equation is described in S1 Text.

**Table 2:** Sensitivity to the model parameters of the final hyphal length reached at 24 h by *Rhizopus oligosporus*.

Parameter	Lower value tested	Higher value tested	Percentage variation in length at 24 h (*F*)
*λ* (μm)	100	600	17%
*k*_*c*_ (g-vesicles h^-1^)	1.4×10^-8^	9×10^-8^	<1%
*K*_*C*_ (g-vesicles dm^-3^)	1	1000	<1%
*k*_*p*_ (g-vesicles dm^-3^ h^-1^)	10	2000	5750%
*K*_*P*_ (g-nutrient dm^-3^)	0.1	50	15%
*m* (g-nutrient g-biomass^-1^ h^-1^)	1.8×10^-4^	1.8×10^-2^	63%
*ω*_0_ (g-nutrient dm^-3^)	1	15	408%
*v* (dm h^-1^)	0	0.08	149%
*ψ* (dm h^-1^)	0.036	0.36	<1%

## Results

### Calibration of the model for the three fungi

**Fig 3:**
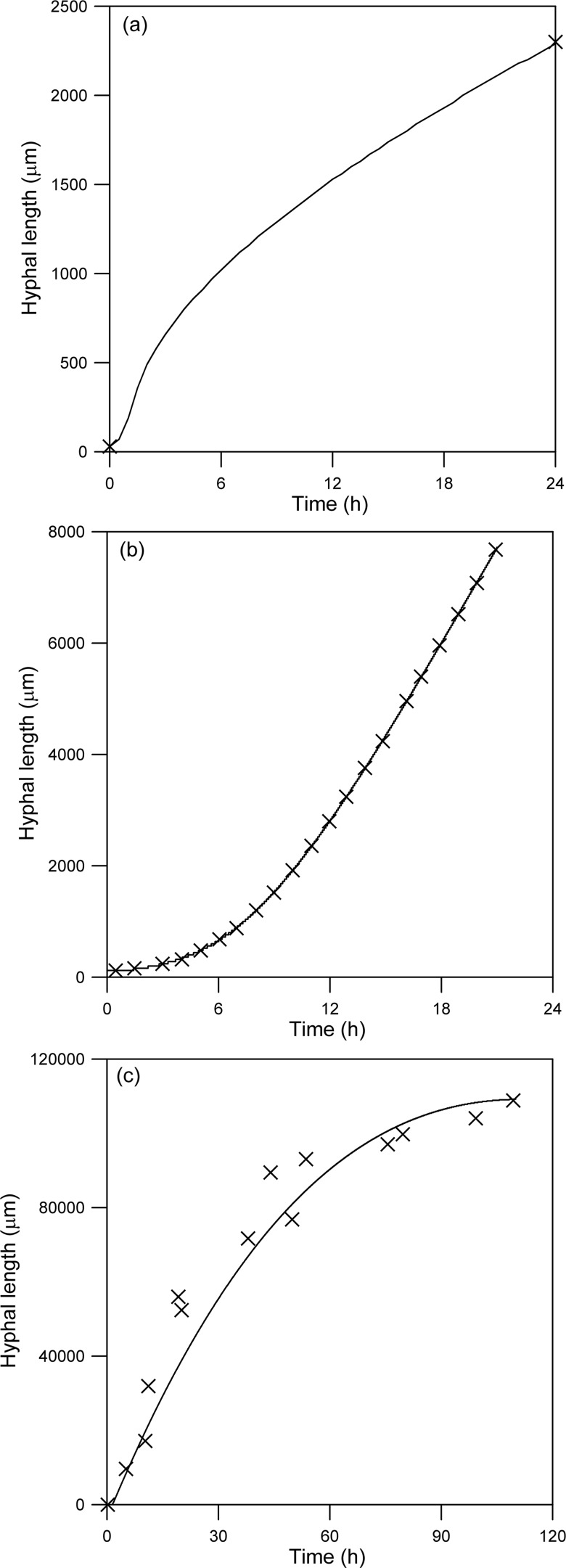
Temporal profile for hyphal extension predicted by the calibrated model. The initial values of the variables and the parameter values are as listed in Table 1. Key: (—) Model predictions; (×) Experimental data. (A) Growth of a sporangiophore of *Rhizopus oligosporus*, data from Nopharatana et al. [13]; (B) Growth of a tall conidiophore of *Aspergillus giganteus*, data from Trinci and Banbury [14]; (C) Growth of a sporangiophore of *Phycomyces blakesleeanus*, data from Gruen [15]. In this particular case, zero time and zero length correspond to 20 h and the hyphal length at 20 h, respectively, in the graph of Gruen [15].

Fig. 3 shows simulated profiles for the extension of reproductive aerial hyphae of *Rhizopus oligosporus*, *Aspergillus giganteus* and *Phycomyces blakesleeanus*. Since Nopharatana et al. [13] only provided confocal images for the growth of the aerial hyphae of *R*. *oligosporus* at 16 and 40 h after inoculation, it is not possible to know the extension profile at intermediate times. In the latter two cases, for which detailed experimental results are available from Trinci and Banbury [14] and Gruen [15], respectively, the model adjusts well. These simulations show that the model is capable of predicting quite different extension profiles: Early acceleration followed by later deceleration (Fig. 3A); initial acceleration for an extended period, followed by linear growth (Fig. 3B); and continuous deceleration (Fig. 3C).

### Investigation of the model predictions

**Fig 4:**
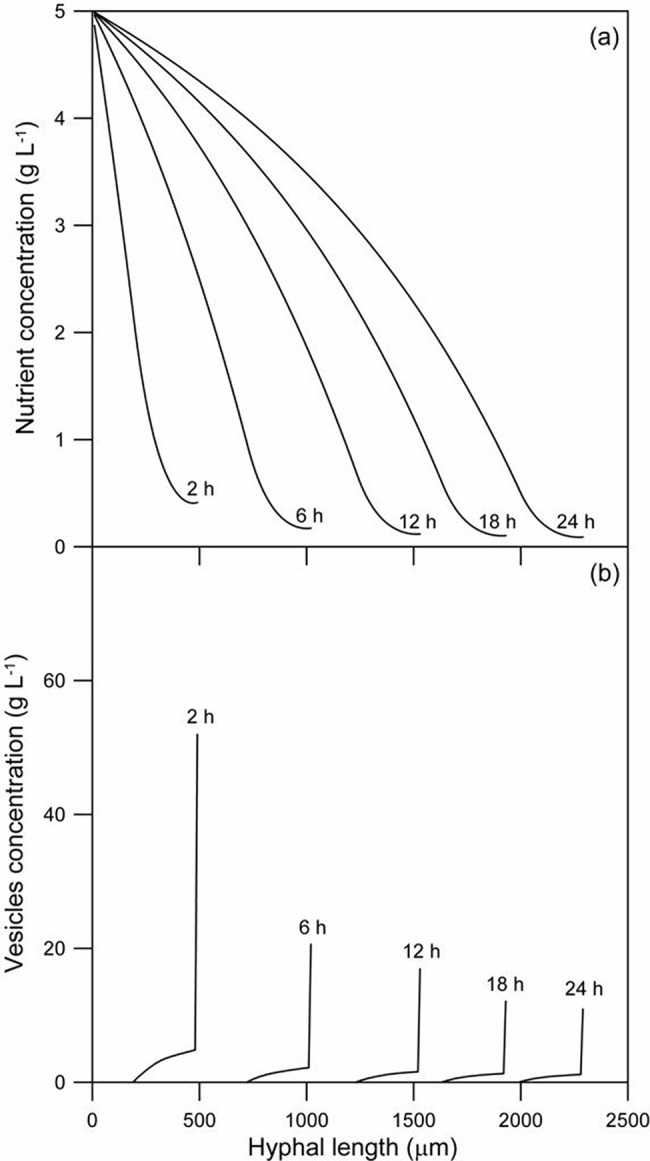
Model predictions for spatial profiles of nutrient and vesicles at different times for *Rhizopus oligosporus*. (A) Nutrient concentration; (B) Concentration of vesicles. The time of each spatial profile is indicated directly on the graph. The initial values of the variables and the parameter values are as listed in Table 1.

The predictions of the model about the processes occurring inside the aerial hypha of *R*. *oligosporus* were explored. Fig. 4A presents the profile of nutrient concentration as a function of hyphal length, at several different times. With increase in time, the curve is longer because the hypha is longer. The concentration of nutrient decreases along the length of the hypha, from the base to the tip, due to the consumption for cellular maintenance and vesicle production. In the region close to the tip, the intense production of vesicles consumes most of the nutrient, resulting in quite low concentrations.

The vesicle concentration as a function of hyphal length is shown in Fig. 4B for various different times. Since vesicles are only produced in the 30 tanks behind the tip, the vesicle concentration is zero 310 μm behind the tip. The vesicle concentration at the tip decreases with time because the concentration of nutrient in the vesicle-producing zone decreases as the hypha elongates (Fig. 4A).

**Fig 5:**
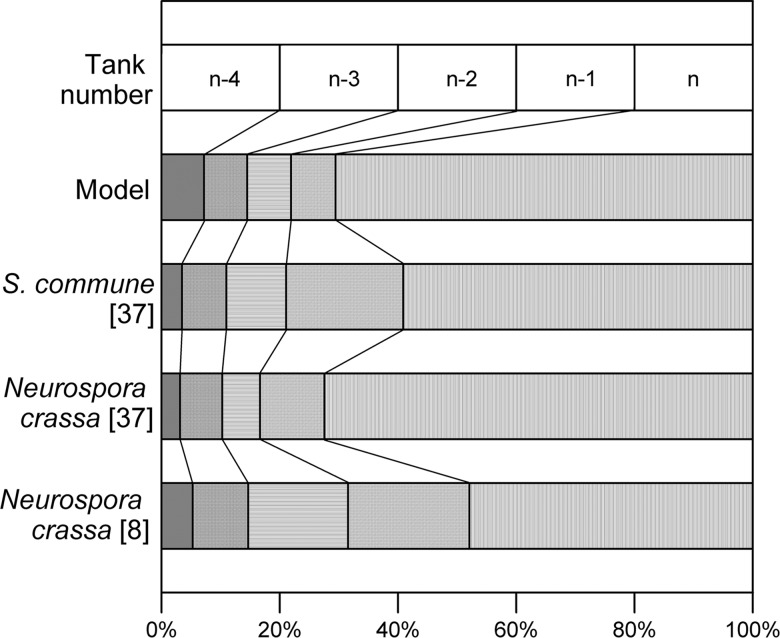
Comparison of the model predictions about vesicle distribution near the tip with experimental data. Model predictions are for 24 h of cultivation, from the simulation done for *Rhizopus oligosporus*. Experimental studies of Collinge and Trinci [8] and Gooday [37] were used for comparison. The experimental data are plotted based on segments of 10 μm length, in order to enable a comparison with the last five tanks of the model.

There is a relatively slow rise in vesicle concentration as the tip is approached and then a marked increase in vesicle concentration in the tip itself. This profile is caused by the active transport of vesicles to the tip through a vesicle-producing zone and is consistent with the profile obtained by Collinge and Trinci [8] for *Neurospora crassa* and by Gooday [37] for *Neurospora crassa* and *Schizophyllum commune*. Integration of their profiles for the volume occupied by vesicles over the 50 μm nearest to the tip shows that 48% to 72% of all the vesicles are present within a distance of 10 μm behind the tip, decreasing to 11% to 21% in the region between 10 and 20 μm behind the tip (Fig. 5). In comparison, the model predicted that, over the 24 h period, between 32% and 72% of the vesicles are in the tip-tank (i.e. between 0 and 10 μm) and between 3% and 8% are in the tank behind the tip-tank (i.e. between 10 and 20 μm).

### Sensitivity analysis

A sensitivity analysis was undertaken in order to determine which of the model parameters have most influence on the length of the aerial hypha of *R*. *oligosporus* after 24 h of growth. The parameters selected for this analysis are listed in Table 2, and are mainly related to transport or to Michaelis-Menten-type expressions (i.e. maximum rates and saturation constants).

The effects of the parameters *K*_*P*_, *λ*, *ψ*, *K*_*C*_ and *k*_*c*_ on hyphal length at 24 h are insignificant (Table 2). An increase in *K*_*P*_ results in a decrease in the rate of vesicle production for a given nutrient concentration. This decrease in the region of the vesicle-producing zone that is furthest from the tip allows more nutrient to reach the vesicle-producing tanks nearer to the tip. The higher nutrient concentration in these nearer tanks leads to a higher rate of vesicle production in this region, such that the overall rate of vesicle production within the vesicle-producing zone remains relatively constant. As a result, the length of the hypha at 24 h is almost unchanged. Changes in the maximum length of the vesicle-producing zone (*λ*) have a similar effect: the distribution of vesicles within the hypha changes, but the hyphal length reached at 24 h is only slightly affected. Parameter *k*_*c*_ has little effect on the hyphal length at 24 h because higher values of *k*_*c*_ lead to lower values for the vesicle concentration in the tip-tank, such that the value of the vesicle consumption term changes little.

**Fig 6:**
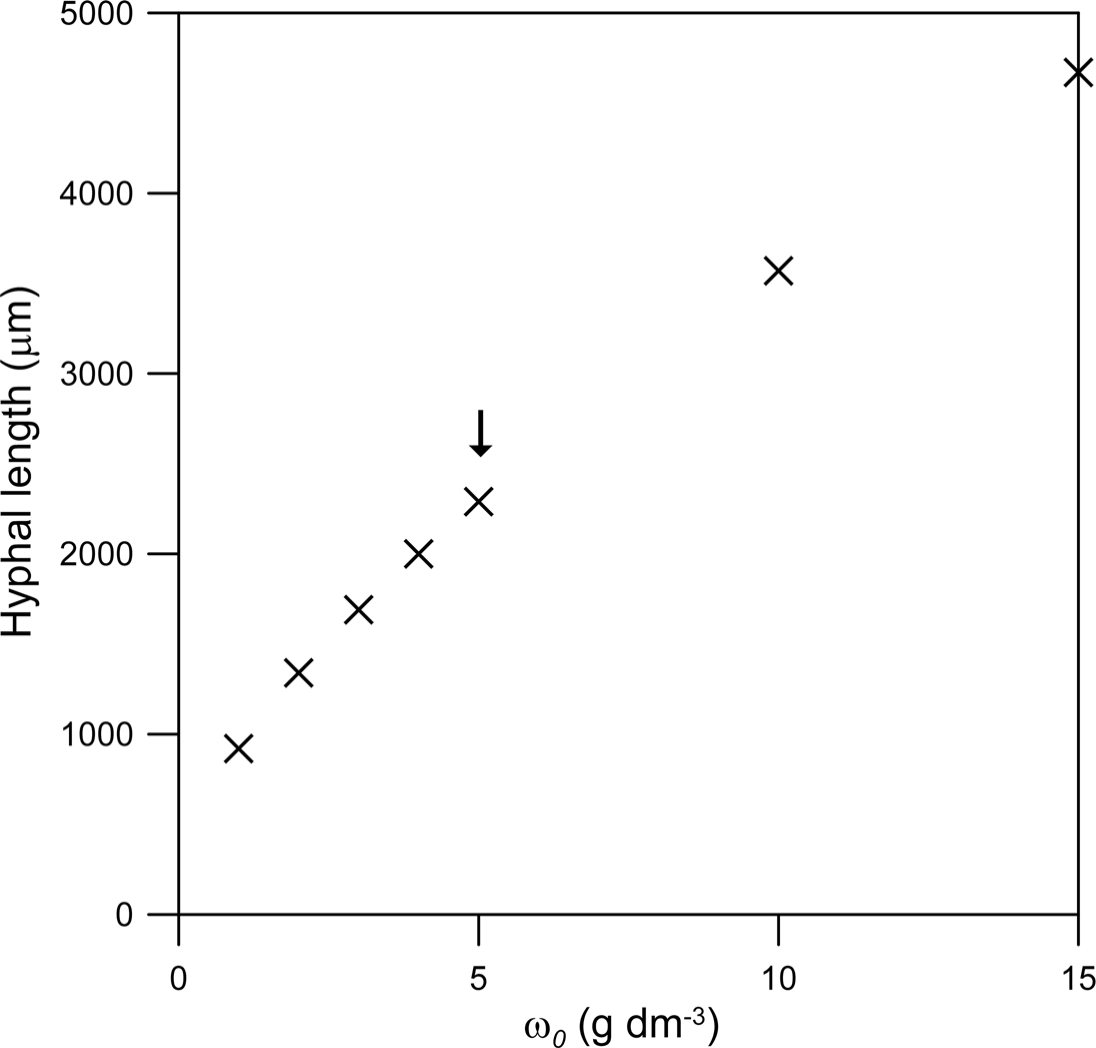
Effect of the concentration of nutrients in the source tank (*ω*_0_). The comparison is based on the hyphal length reached at 24 h. The original value (i.e. that used for Figs. 3A and 4) is indicated with an arrow. The initial values of the variables and the other parameter values are as listed for *Rhizopus oligosporus* in Table 1.

**Fig 7:**
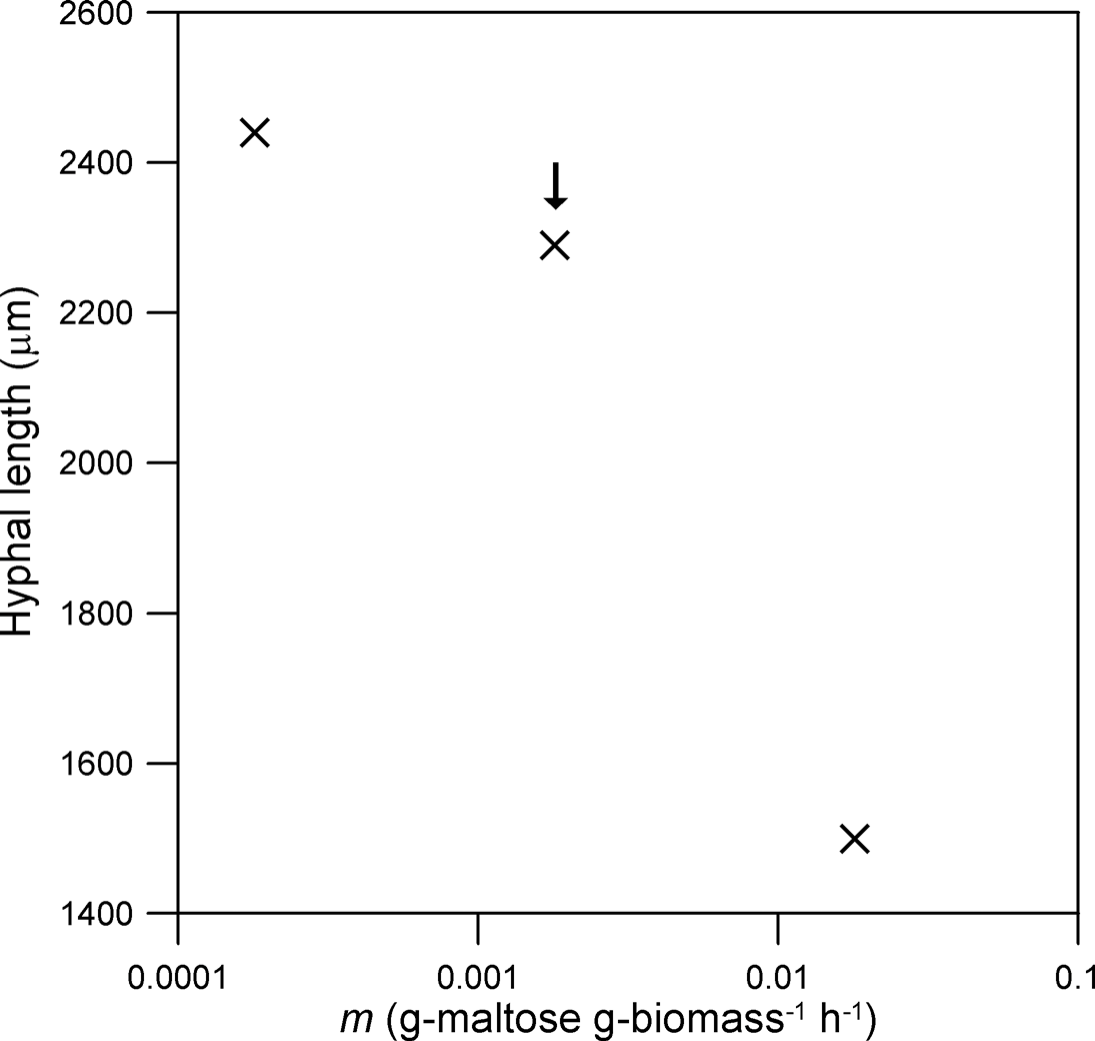
Effect of the maintenance coefficient (*m*). The comparison is based on the hyphal length reached at 24 h. The original value (i.e. that used for Figs. 3A and 4) is indicated with an arrow. The initial values of the variables and the other parameter values are as listed for *Rhizopus oligosporus* in Table 1.

**Fig 8:**
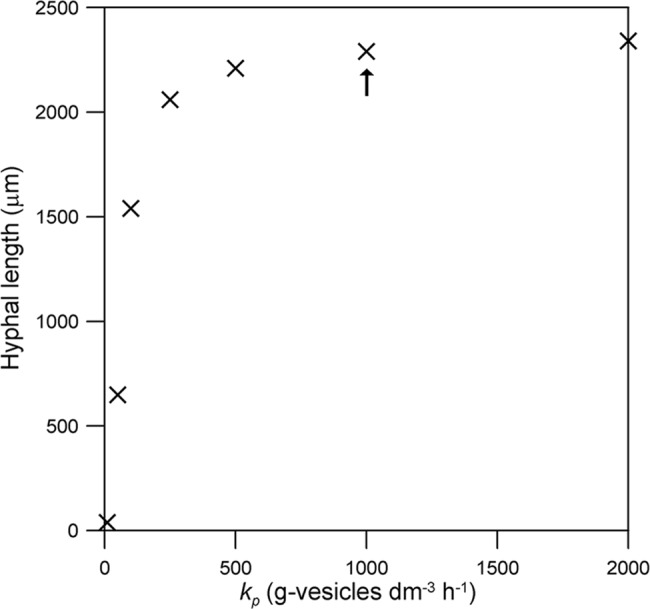
Effect of the maximum rate of vesicle production (*k*_p_). The comparison is based on the hyphal length reached at 24 h. The original value (i.e. that used for Figs. 3A and 4) is indicated with an arrow. The initial values of the variables and the other parameter values are as listed for *Rhizopus oligosporus* in Table 1.

The parameters with the greatest effect on the rate of extension of the hypha are those that affect the supply of nutrient to the vesicle-producing zone: *ω*_0_, *v*, *m* and *k*_*p*_. Over the range tested, the hyphal length at 24 h is directly proportional to *ω*_0_ (Fig. 6). The effect of the maintenance coefficient (*m*) is less pronounced (Fig. 7). For example, in relation to the value listed in Table 1, a 10-fold decrease in *m* increases the hyphal length at 24 h by only 7%, while a 10-fold increase in *m* causes this length to fall by 65% (Fig. 7). The dependence of the hyphal length at 24 h on *k*_*p*_ has a profile similar to a Michaelis-Menten curve, with a large effect at low values of *k*_*p*_ but very little effect at high values of *k*_*p*_ (Fig. 8).

**Fig 9:**
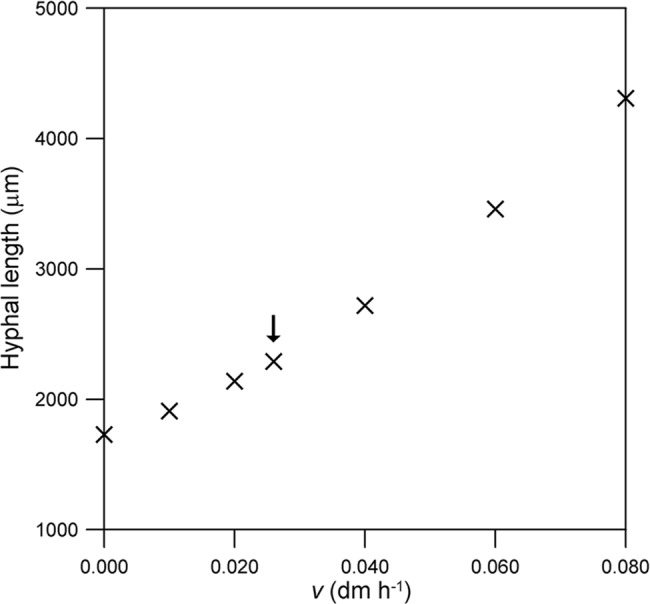
Effect of the convective flow velocity within the hypha (*v*). The comparison is based on the hyphal length reached at 24 h. The original value (i.e. that used for Figs. 3A and 4) is indicated with an arrow. The initial values of the variables and the other parameter values are as listed for *Rhizopus oligosporus* in Table 1.

The hyphal length reached after 24 h of growth is significant (1730 μm) in the absence of intracellular convection (*v* = 0) and increases exponentially over the interval of *v* tested (Fig. 9). The result for *v* = 0 is hypothetical, since intracellular convection is essential to supply cytoplasm to the new tip in aerial reproductive hyphae, but it does demonstrate that diffusion makes a significant contribution to the transport of nutrient within the hypha.

## Discussion

The model developed in the current work is the first discrete intermediate-scale model of hyphal extension to describe not only the transport of a soluble nutrient within the hypha but also the presence of a defined vesicle-producing region behind the tip that converts the nutrient into vesicles that are then transported to the tip. A key aspect of this work is the use of the n-tanks-in-series approach to describe this system. This approach enables the model to describe, in a relatively simple manner, the fact that different regions of the hypha have different physiological processes. Although our model is relatively simple, it has the flexibility to be extended to describe phenomena that are important in the development of real mycelia. With the incorporation of these modifications, it will be suitable for fusing with the spatial model of Coradin et al. [25] to produce a model that can describe how the development of a fungal mycelium in three dimensions is affected by intracellular transport of nutrients and vesicles. The following sections discuss these points, as well as insights that our modeling work has given into the growth of reproductive aerial hyphae.

### Insights into the growth of reproductive aerial hyphae

Although the main aim of the current work was to develop a hyphal extension model based on the role of vesicles and our decision to use our model to describe the growth of reproductive aerial hyphae was based on the simplicity of this system, the model predictions raise some important questions.

For an aerial hypha, the nutrients for tip extension must necessarily be provided by the cells at the base of the hypha. This is different from the case of surface and penetrative hyphae, which are in intimate contact with the substrate and can absorb nutrients through their side walls along the whole length of the hypha. Our sensitivity analysis suggests that a major factor controlling the rate of extension of a reproductive aerial hypha is the rate at which nutrients are delivered to the vesicle-producing zone. This rate of delivery depends on the nutrient concentration in the source tank (i.e. in the vegetative hypha), the convective velocity with which nutrient is swept along the hypha and nutrient consumption for maintenance throughout the hypha. These phenomena are not well studied for aerial hyphae; their importance in the simulations suggests that they deserve attention in the future. Although cytoplasmic streaming, which is responsible for the convective flow of soluble species within the hypha, is well known, reported rates are mostly for surface hyphae. Furthermore, it is essential to have more experimental data on the physiology of the vegetative hypha attached to the reproductive hypha, such as the production of glycogen and its later hydrolysis. This would allow modeling of the processes that provide the carbon source at the base of the aerial hypha.

Experimental determinations of the length of the vesicle-producing zone in aerial hyphae are also necessary. The only study of vesicle production in aerial hyphae is for *Aspergillus nidulans*, for which the vesicles are present throughout the entire 100-μm length of the aerial hypha [18], whereas many fungi have aerial hyphae varying from a few millimeters to several centimeters in length. Also, in our model there is no transfer of vesicles from the vegetative hypha to the reproductive hypha because the micrographs of Mims et al. [18] do not show vesicles in the vegetative hypha of *A*. *nidulans*, however, for other fungi the vegetative hyphae might supply vesicles to nascent reproductive hyphae.

### Comparison with previous discrete intermediate-scale models that describe vesicles

Our model describes the production, transport and consumption of vesicles in a more realistic manner than previous discrete intermediate-scale models do. The models of Prosser and Trinci [7] and Yang et al. [11] not only omit a description of the production of vesicles from intracellular nutrient, but also have other limitations. In both models, the production of vesicles is not restricted to a particular region of the hypha, rather it occurs at a constant rate along the whole hypha, regardless of its length. Further, in the model of Yang et al. [11], not only do the vesicles move solely by diffusion, but the concentration of vesicles at the tip is set to zero, with the rate of extension being directly proportional to the rate at which vesicles reach the tip. This zero concentration of vesicles in the tip is not observed experimentally (see Fig. 5).

The only previous discrete intermediate-scale model that describes the production of vesicles from a nutrient source is that of López-Isunza et al. [12]. Their assumption that the whole hyphal length contributes vesicles for the extension at the tip may be appropriate for a germ tube, but means that, unlike our model, their model is not able to simulate differently shaped growth profiles; rather it always predicts a continuous acceleration. In any case, their assumption would not be appropriate for long hyphae, in which only the vesicle-producing zone immediately behind the tip contributes [29]. Their model also assumes that macrovesicle transport has a diffusive component, whereas recent evidence shows that macrovesicle transport is a unidirectional active process [24]. Further, their model is not fully predictive, as it requires the final length of the germ tube as input.

Our model is the first model to recognize a defined vesicle-producing zone. The existence of this zone, which represents the length of the hypha that contributes to hyphal extension, has been known since 1971 [29]. As a result of the fact that our model describes vesicle production and transport more realistically than previous models do, it is the first model that is able to reproduce the peak in vesicle concentration at the tip that is observed experimentally (Fig. 5). In contrast, the model of López-Isunza et al. [12] predicts a decrease in the concentration of vesicles along the hypha in the direction of the tip.

### Advantages of the n-tanks-in-series approach

Our model represents the first time that the n-tanks-in-series approach has been applied to describe how the extension of a fungal hypha depends on the supply of nutrients and vesicles. This approach is mathematically simpler than the approach of treating the hypha as a single continuous domain over its whole length, as was done by Yang et al. [11], López-Isunza et al. [12] and Boswell et al. [26]. In a continuous system, the extension of the hyphal tip means that the spatial domain for integration needs to be increased in length. This, in itself, represents a challenge and requires the implementation of specialized numerical techniques [26]. With the n-tanks-in-series approach, the extension of the hypha is quite easily described without the need for these specialized techniques. Additionally, with the n-tanks-in-series approach, it is a simple matter to represent the processes at the tip. In a continuous system, a boundary condition at the tip must be specified for each partial differential equation. For an individual hypha this is not difficult, however, it becomes problematic when modeling various tips within a mycelium.

Further, the n-tanks-in-series approach has a much greater flexibility to describe the spatial and temporal complexity of real hyphae. For example, with this approach, it is easy both to describe different physiological processes occurring simultaneously in different regions of a hypha and to change the physiological processes that occur in a particular region of the hypha over time. The particular case that was demonstrated in the current work is the presence of a sub-apical vesicle-producing zone that moves behind the tip, such that any given tank is first created as a tip-tank, is then converted into a vesicle-producing tank and then later loses its ability to produce vesicles as the tip extends away from it. This approach could be extended to describe any type of physiological differentiation within a hypha. It would be too complex to describe this behavior using a continuous model that uses partial differential equations.

### Flexibility of model to incorporate other phenomena involved in mycelium development

Although the model presented in the current work describes a relatively simple system, namely the extension of a single reproductive aerial hypha, with nutrients being supplied solely by a vegetative hypha at its base, the model was developed to be sufficiently flexible for it to be possible to incorporate descriptions of a wide range of phenomena that occur during the growth of mycelia of filamentous fungi in real systems. Some possible extensions of the model are described below.

Firstly, the model could be used to describe the formation of a branched mycelium, such as that modeled by Coradin et al. [25]. Depending on the fungal species being modeled, the branching would be apical or sub-apical. Of course, it would be necessary to describe the division of flow and mass transfer at branch points [7].

Secondly, the model could be used to describe the formation of septa. The current model describes an aseptate hypha, thus the boundaries of the tanks are not barriers to flow or diffusion. The boundaries between specific tanks could be made to represent septa. Of course, since the distance between septa is usually greater than 10 μm, not all inter-tank boundaries would be designated as septa. Since most septa are incomplete divisions, they will diminish transport, but not stop it [29]. The formation of a septum would require a change within the equations that describe transport between the particular tanks separated by that septum. Since in septate fungi the accumulation of vesicles behind a septum in a sub-apical compartment often is the trigger for the initiation of sub-apical branches [38], it is important for models of mycelial growth to describe the production and transport of vesicles.

Thirdly, although at the moment all the water and nutrients that enter the reproductive aerial hypha are supplied from the vegetative hypha to which it is attached, in order to describe surface and penetrative hyphae, the model could be extended to describe the absorption of water and nutrients through the side walls of the tanks by incorporating appropriate terms into the corresponding balance equations [12,26].

Finally, a more complete model of the whole mycelium (penetrative, surface and aerial hyphae) might involve the closing off of older hyphae and even autolysis of certain regions (i.e. the disappearance of tanks) [26].

### Towards a 3-D mycelial growth model including transport mechanisms

To date, the most appropriate model for describing the development of a fungal mycelium in a system containing spatially complex arrangements of solids and air is that of Boswell et al. [39,40]. It describes the phenomena of intracellular diffusion and convection of nutrients, branching and anastomosis (i.e. when a tip fuses with an existing hypha after contact) and was used to describe the development of fungi amongst soil particles. However, even though the model dealt with vegetative hyphae of *Rhizoctonia solani*, which have a diameter of about 10 μm [41], these hyphae were assumed to extend in 100 μm steps, therefore, the model is not able to describe phenomena that occur at smaller scales. For example, the intervals between branches can be as little as 20 μm [42] and, for penetrative hyphae, the dissolved O_2_ concentration can fall to zero for depths as small as 100 μm below the gas-liquid interface [43]. Another disadvantage is that the hyphae were confined to move within a regular hexagonal lattice, and therefore the simulated mycelium did not have the features of a real mycelium.

An alternative approach to modeling mycelial growth in three dimensions is that of Coradin et al. [25]. The mycelium is represented by interconnected cubes of 10 μm a side, which is of the order of magnitude of the diameter of many hyphae. The model describes tip elongation and branching to produce new tips. It contains rules for the choice of the direction of extension of each tip and either avoidance or inactivation if a tip contacts an existing hypha. However, it does not describe the transport of nutrients within the hyphae, rather it considers tip extension as an automatic process that occurs with each iteration of the model.

The model developed in the current work, after the incorporation of the phenomena discussed in the previous subsection, will provide the basis for incorporating mass transfer phenomena into the spatial model of Coradin et al. [25]. Once this is done, it will be possible to describe the growth of a mycelium in systems that involve the development of aerial, surface and penetrative hyphae on particles of solid material. As pointed out by Coradin et al. [25], the 10 μm grid size gives the model a flexibility to describe morphological features of real mycelia at microscopic scales similar to that which is possible with lattice-free models, while making the task of checking for intersections quite simple, as the biomass occupies discrete cubes.

The relatively small grid size of the model would lead to overly demanding computational requirements if this model were to be used to describe the growth of filamentous fungi at the macro-scale, but it could be a useful tool for modeling at scales of the order of a few cubic millimeters [25]. Models capable of describing what happens at this scale could be used to investigate the key growth and transport phenomena in systems containing solid substrates in contact with an air phase, such as the growth of penetrative hyphae into the solid support, the changes in the solid support due to nutrient consumption by the hyphae, the growth of aerial hyphae into the inter-particle spaces and particle-to-particle colonization.

## Conclusion

We have developed the first model for hyphal growth that combines a description of nutrient transport with the production of vesicles in a defined sub-apical vesicle-producing zone. Although in the current work we used our model to describe the extension of a single reproductive aerial hypha, the use of the n-tanks-in-series approach means that it has the flexibility to be extended to describe the extension of surface and penetrative hyphae and branching to form a mycelium. As such, it is appropriate to fuse with the model of Coradin et al. [25] to describe the growth of fungal mycelia in spatially complex arrangements of solids and air. A further advantage of this approach is that it simplifies the description of changes in physiological states, not only with position along a hypha but also over the time course of growth. Our simulations suggest that the extension of a reproductive aerial hypha is mainly influenced by the rate of supply of the nutrient to the vesicle-producing region, which, in turn, depends on the concentration of nutrient at the base of the hypha, the convective velocity of nutrient within the hypha and nutrient consumption for maintenance. These phenomena deserve further attention since they are not well studied in reproductive hyphae.

## Supporting Information

S1 Text The algorithm for tank indexing. (DOC)
