# Supplementary material for: A Model for Growth of a Single Fungal Hypha Based on Well-Mixed Tanks in Series: Simulation of Nutrient and Vesicle Transport in Aerial Reproductive Hyphae
Source: PLoS One. 2015 Mar 18;10(3):e0120307. doi: 10.1371/journal.pone.0120307 (PMC4364911; doi:10.1371/journal.pone.0120307)
Supplement: S1 Text — (DOC) [file pone.0120307.s001.doc]

**S1 Text. The algorithm for tank indexing**

The model describes the extension of a single reproductive aerial hypha and the concentration of nutrient and vesicles along the hyphal length. The hyphal length is divided into tanks numbered from 1 to *n*. The index of a particular tank does not change during the simulation, hence tank 1 will always be the tank closest to the vegetative hypha (i.e. closest to the source tank) and tank *n* will always be the tip-tank. Each time that a new tank is added through division of the tip-tank, *n* increases by 1.

Figure S1 shows the hyphal tanks with their corresponding tank numbers and corresponding equations for temporal variation of the nutrient and vesicle concentrations.

The concentrations of nutrient and vesicles in each tank are described by a pair of ordinary differential equations (ODEs). The extension of the tip-tank is also described by an ODE. Therefore, the number of ODEs that need to be solved (K) depends on the number of tanks (*n*):

$$K=2n+1$$

When the length of the tip-tank reaches 2Δx (i.e. twice the length of a “normal” tank), the tip-tank divides into two tanks: a tip-tank (index *n*) and an intermediary vesicle producing tank (index *n*-1). Since the number of tanks increases (i.e. *n* increases by 1 during the act of division) the number of ODEs also increases.

extension of
tip-tank = Eq (5)

**Representation of the hypha by tanks, for a hypha 40 tanks long, indicating the equations applying to each tank**

Tank 1

y’(A_1,1_) = Eq. (1)

y’(A_1,2_) = Eq. (2)

Tank 2

y’(A_2,1_) = Eq. (3)*

y’(A_2,2_) = Eq. (4)*

Tank 3

y’(A_3,1_) = Eq. (3)*

y’(A_3,2_) = Eq. (4)*

Tank 38 (*n*-2)

y’(A_38,1_) = Eq. (3)

y’(A_38,2_) = Eq. (4)

Tank 39 (*n*-1)

y’(A_39,1_) = Eq. (3)

y’(A_39,2_) = Eq. (4)

Tank 40 (tank *n*)

y’(A_40,1_) = Eq. (6)

y’(A_40,2_) = Eq. (7)

Equations

for nutrient →

Equations

for vesicles →

**Y vector integrated by DASSL**

*the symbol “*A_B_,_C_*“ in a tank above represents the value contained on line B and in column C of matrix A below.*

1

Length of tip-tank

[nutrient] in tank 1

[vesicles] in tank 1

2

3

**Matrix A (matrix of addresses)**

4

[nutrient] in tank 2

Column 1

[nutrient]

Line 1 = Tank 1

Line 2 = Tank 2

Line 3 = Tank 3

Line 38 = Tank 38

Line 39 = Tank 39

Line 40 = Tank 40

Column 2

[Vesicles]

2

3

4

5

6

7

76

77

78

79

80

81

etc

etc

etc

[vesicles] in tank 2

5

[nutrient] in tank 3

6

[vesicles] in tank 3

7

*The value contained in matrix A represents the address at which the corresponding variable is to be found within the Y vector that is integrated by the subroutine DASSL*

etc...

[nutrient] in tank 38

76

[vesicles] in tank 40

[vesicles] in tank 38

77

...etc

78

[nutrient] in tank 39

[vesicles] in tank 39

79

Figure S1. Representation of the hyphal tanks with their corresponding tank numbers and corresponding equations for temporal variation of the nutrient and vesicle concentrations. Representations correspond to the simulation when there are 40 tanks. Equation numbers refer to the numbers used in the main document, those marked with an asterisk (*) do not contain the term for vesicle production.

[nutrient] in tank 40

80

81

All ODEs are solved simultaneously using the DASSL routine. The DASSL routine requires that all the values of all derivatives be grouped into a single vector: YPRIME(*K*). The corresponding variables must also be grouped into a single vector: Y(*K*). Hence, the concentration of vesicles and nutrient in each tank and the length of the tip-tank are written in a single vector, Y, in the algorithm, in a contiguous manner. The lengths of vectors YPRIME and Y correspond to the number of ODEs to be solved (*K*). The index for a given variable in vector Y is the same index of the corresponding ODE in vector YPRIME.

As each tank is formed, it receives one address for nutrient concentration and one for vesicle concentration, these addresses indicating where the value of the corresponding variable/derivative is to be found in vectors Y and YPRIME. These addresses are recorded in an auxiliary matrix A (size *n ×* 2) which is shown in Figure S1: column 1 contains the addresses that correspond to nutrient concentrations; column 2 contains the addresses that correspond to vesicle concentrations; and each line represents the number of the tank to which each variable refers. For example, the address in vector Y for nutrient concentration in tank four is 8 and this address is recorded in A_4,1_. In other words, Y(A_4,1_) is the nutrient concentration in tank four and YPRIME(A_4,1_) is the derivative of nutrient concentration in tank four. Similarly, Y(A_1,2_) is the vesicle concentration in tank 1. Given that there is only one ODE for the length of the tip-tank, this variable is recorded in Y(1). The addresses recorded in matrix A are assigned as each tank is created and, once assigned, they are not changed as the simulation proceeds.

The algorithm for the solution of the model is represented in Figure S2. The ODE for each tank depends on the tank number: Eqs. (1) and (2) are used for the first tank, while Eqs. (6) and (7) are used to describe the tip-tank (tank *n*) and Eqs. (3) and (4) are used for the intermediate tanks (tanks 2 to *n*-1) (equation numbers refer to the numbers used in the main document). According to the position relative to the tip-tank and the length of the vesicle-producing zone, the first tank and the intermediate tanks may be vesicle-producing tanks or not. For example, in the simulation with *Rhizopus oligosporus*, since *N_V_* = 30, if there are less than 30 tanks between a given tank and the tip-tank, then that tank is a “vesicle-producing tank”. If there are 30 tanks or more between a given tank and the tip-tank, then that tank does not produce vesicles, thus, the vesicle production term must be eliminated from Eqs. (1) to (4). The use of matrix A also allows the tracking of adjacent tanks, which facilitates the implementation of Eqs. (1) to (7).

After each iteration, the length of the tip-tank is evaluated to test if it will divide or not. If the tip-tank is shorter than double the size of a normal tank, than the initial conditions for the DASSL routine are updated and the equations are solved for another small time step. However, if the tip-tank is equal to or longer than double the size of a normal tank, the tip-tank divides, at which instant *n* is increased by 1. The creation of a new tank requires that matrix A be updated.

Figure S2. The algorithm used to solve the model. Equation numbers refer to the numbers used in the main document, those with * are those that do not contain the term for vesicle production.
